# Eosinophilic meningitis caused by *Angiostrongylus cantonensis* in an infant: A case report

**DOI:** 10.1097/MD.0000000000010975

**Published:** 2018-06-15

**Authors:** Mingsheng Ma, Mengqi Zhang, Zhengqing Qiu

**Affiliations:** Department of Pediatrics, Peking Union Medical College Hospital, Chinese Academy of Medical Sciences and Peking Union Medical College, Beijing, China.

**Keywords:** eosinophil, fever, meningitis

## Abstract

**Rationale:**

Parasitic eosinophilic meningitis is rarely observed in infants. The diagnosis of this disease is complicated by its atypical and severe clinical manifestations.

**Patient concerns:**

An infant presented to our hospital with high fever and irritability, as well as refusal to walk. Cerebrospinal fluid collected through lumbar puncture showed increased eosinophil count and third-stage *Angiostrongylus cantonensis* larvae.

**Diagnoses:**

Eosinophilic meningitis was suspected.

**Interventions:**

We started empiric treatment with levamisole (14 mg bid, 2.5 mg/kg·day) and prednisone (17.5 mg qd, 1.5 mg/kg·day).

**Outcomes:**

All of the infant's symptoms were resolved approximately 72 hours after treatment. The patient fully recovered from her illness after completing 4 weeks of levamisole and prednisolone treatment.

**Lessons:**

*A. cantonensis* is the most common cause of parasitic eosinophilic meningitis cases in Southeast Asia. Physicians treating infants who live in areas where *A. cantonensis* is endemic and who present with irritability, abnormal motor function, and elevated eosinophil count should be aware of the disease to provide timely and rational therapy to the patients.

## 1 Introduction

*Angiostrongylus cantonensis* is the most common cause of eosinophilic meningitis among humans living in Southeast Asia and throughout the Pacific Basin. Individuals become infected with this parasite by consuming contaminated raw snails, vegetables, small mollusks, or fresh water. The third-stage larvae of *A cantonensis* induce eosinophilic meningitis once entrenched in the central nervous system. Eosinophilic meningitis induced by *A cantonensis* infection is rarely observed in infants.^[1]^ Furthermore, the diagnosis of this disease is complicated by its atypical and relatively severe clinical manifestations. We present a case of a 15-month-old Chinese girl with eosinophilic meningoencephalitis caused by *A cantonensis*. Moreover, we review the methods used to diagnose and examine this disease in infants.

## 2 Ethical statement

Given that this study mainly involves retrospective patient observations, ethical approval was unnecessary. Informed consent was obtained from the patient's father for publication.

## 3 Case report

A 15-month-old girl was admitted to the Peking Union Medical College Hospital (PUMCH). The girl had experienced high fever for 3 weeks, irritability for 2 weeks, and refusal to walk for 1 week. Three weeks before admission to PUMCH, she was seen at a local clinic (Haikou City, Hainan Island, located in the southern end of China) for fever and constipation. Physical examination was normal, and she was treated with ibuprofen. One week later, she presented to Haikou People's Hospital with a persistent high fever of over 39.5°C, irritability, and crying during the night. The result of head computer tomography scan was normal, and lumbar puncture revealed an opening pressure of 140 mm H_2_O. Her cerebrospinal fluid (CSF) was clear and had 120 × 10^6^/L white blood cells with 38% neutrophils and 62% lymphocytes. The patient was treated for viral meningitis with an antiviral for 2 weeks. Thereafter, she refused to walk because of lower limb pain. The patient's fever was not relieved by treatment.

This girl was gravida 1 para 1 and was the full-term baby of an uncomplicated pregnancy. She had been raised in the countryside of Hainan Island. She had normal developmental milestones, uneventful previous history, and full immunization for her age.

The patient cried and was extremely irritable during the physical examination. The physical examination showed that she had a weight of 11.5 kg and a temperature of 40°C. Rashes, lymphadenectasis, and joint redness were not observed. Skin sensation could not be evaluated because the patient responded to any skin contact with exaggeration and crying. The patient's muscle strength and tone were normal even though she refused to stand or walk. The jerk reflexes of her limbs were symmetrical, and her pathological reflex was negative. No obvious focal neurologic signs were detected.

Laboratory tests showed a peripheral blood leukocyte count of 12.6 × 10^9^/L with an increased eosinophil count of 21.3% and absolute eosinophil count of 2.69 × 10^9^/L. The results of renal and liver function tests and routine urine and stool tests were normal. Serum IgM antibodies to cytomegalovirus, herpes virus simplex, and Epstein-Barr virus were negative.

**Figure 1:**
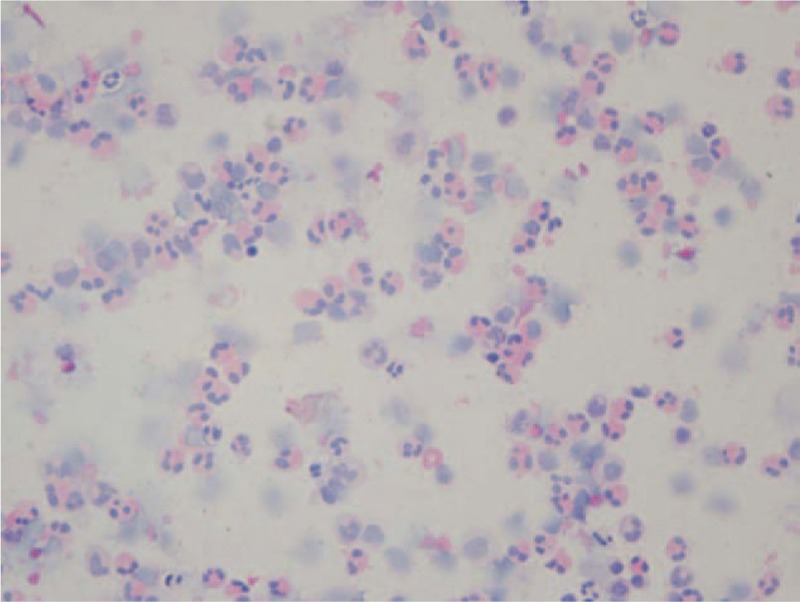
An image of hematoxylin and eosin staining of the patient's CSF (40×).

The magnetic resonance image of her brain was normal. Lumbar puncture disclosed an opening pressure of 200 mm H_2_O, and the CSF appeared cloudy. CSF cytology showed a white blood cell count of 400 × 10^6^/L with 70% eosinophils, 32% monocytes, and 3% lymphocytes (Fig. 1). The chemistry test results of the patient's CSF showed 2.2 mmol/L glucose, 122 mmol/L chloride, and 600 mg/dL total protein. The Gram stain, India ink stain, and the acid-fast stain results of the CSF were negative. CSF aerobic and anaerobic cultures and cultures for *Mycobacterium tuberculosis* and fungi were all negative.

Parasitic eosinophilic meningitis was highly suspected because of the patient's increased blood and CSF eosinophil count and persistent neurological symptoms. The mother was further questioned for the infant's exposure history. She recalled seeing the child play with slugs.

Eosinophilic meningitis was diagnosed clinically on the basis of the child's exposure history, and parasite-specific investigations were initiated. The suspected third-stage larvae of *A cantonensis* were found in the CSF. However, a reference laboratory in Beijing Tropical Medical Research Center reported that the patient's serum sample tested negative for antibodies against *A cantonensis*.

Considering the patient's clinical symptoms and signs, increased intracranial pressure and eosinophil levels in peripheral and CSF samples, and lack of response to antiviral treatment, as well as the presence of parasites in CSF, oral empiric treatment with 2.5 mg/(kg/day) levamisole and 1.5 mg/(kg/day) prednisone together with mannose was initiated to reduce intracranial pressure.

The patient exhibited rapid recovery, and all of her symptoms resolved approximately 72 hours after the commencement of treatment. Hematological testing and CSF examination revealed that the child's eosinophil count had decreased. Thus, prednisone dosage was gradually decreased. Another lumbar puncture performed 4 weeks after the first lumbar puncture revealed that the eosinophil levels in the child's CSF had decreased to 0. The patient was discharged from the hospital and completed 4 weeks of treatment with levamisole and prednisolone. At the 6-month follow-up, the patient showed normal growth and development without sequelae.

## 4 Discussion

Eosinophilic meningitis in infants is rare, and its diagnosis is complicated by its atypical clinical manifestation. To the best of our knowledge, this is the first report of *A cantonensis* infection in a Chinese infant. Here, we summarize the epidemiology, clinical presentations, laboratory data, treatment, and clinical outcome of *A cantonensis* infection in an infant.

*A cantonensis* is the most common cause of parasitic eosinophilic meningitis in humans living in Southeast Asia and throughout the Pacific Basin. Previous cases of parasitic eosinophilic meningitis in infants were distributed around Pacific, American Samoan,^[1,2]^ Hawaii,^[3]^ Jamaica,^[4]^ and Australia.^[5]^ Humans become infected with *A cantonensis* by consuming raw snails, vegetables, small mollusks, or fresh water contaminated with the third-stage larvae of this parasite. A history of consuming the intermediate or paratenic hosts of *A cantonensis* is critical for diagnosis. This history, however, is difficult to confirm in infants because of their inability to speak. Infants who indiscriminately place objects in their mouths may ingest infective larvae from various sources. Our patient resided in Hainan Island, which is located in the southern end of China where *A cantonensis* is widely distributed.^[6]^ Our patient's mother recalled that the child had picked up and swallowed a slug. However, in most cases, the source of the infection is not established.^[3–5]^

The main symptoms of eosinophilic meningitis in adults include headache, neck stiffness, paresthesias, vomiting, and nausea.^[7]^ However, the symptoms of infants with eosinophilic meningitis differ greatly from those of adults. Compared with adults, infants are less likely to present headache, neck stiffness, and paresthesias but are more likely to present fever, irritability, and motor-function abnormalities.^[1–5]^ The incidence rates of anorexia and constipation are relatively higher among infants than among adults. Our patient presented fever and refusal to walk. Similar features were observed in 2 other infants.^[2,4]^

The detection of circulating antigens in serum or CSF enables the rapid confirmation of infection. However, the diagnosis of *A cantonensis* in clinically suspected patients should not be excluded on the basis of negative CSF test results.^[8]^ Recent research found that CSF eosinophil counts of 40% or higher are significantly predictive for positive serologic tests.^[9]^ Although the CSF eosinophil levels of most previous infant cases were higher than 40%,^[1–3]^ the diagnosis of *A cantonensis* should not be excluded on the basis of absent CSF eosinophilia.^[5]^

The parasites that were observed in the CSF of our patient were identified as the third-stage larvae of *A cantonensis.* Similarly, parasites have been found in the CSF of a 17-month-old boy.^[4]^ Parasites were also observed in the brains and lungs of 2 fatal cases.^[4,5]^

Corticosteroid therapy has been effective in the treatment of human angiostrongyliasis, and anthelminthics have been used to relieve and shorten the duration of symptoms of this disease. The combination of corticosteroids and anthelminthics has been commonly used to treat human angiostrongyliasis.^[10]^ We treated our patient with prednisone and levamisole. Supportive and symptomatic treatments are also important for infants.^[2,3]^

Most cases with *A cantonensis* are self-limited, and fatal encephalitic angiostrongyliasis among adults is rare. However, infants are more likely to die than adults from encephalitic angiostrongyliasis induced by *A cantonensis* because they cannot be diagnosed rapidly. Two out of 5 previously reported cases of encephalitic angiostrongyliasis were fatal and involved the lungs of the infants.^[4,5]^ Our patient and 2 other previous patients presented good recovery,^[1,3]^ and 1 infant had hydrocephalus.^[2]^

Eosinophilic meningitis in infants is rare. Its diagnosis is complicated by its atypical clinical manifestation and the difficulty in confirming the patient's history of consuming intermediate or paratenic hosts of *A cantonensis*. Physicians treating infants who live in areas where *A cantonensis* is endemic and who present with irritability, abnormal motor function, and elevated eosinophil count should be aware of the disease to provide timely and rational therapy to the patients.

## Author contributions

**Writing – original draft:** Mingsheng Ma.

**Writing – review & editing:** Mengqi Zhang, Zhengqing Qiu.

## References

[1] Maurer DM Greene JP Vincent JM Fever, refusal to walk and eosinophilia in a ten-month-old Samoan boy. Pediatr Infect Dis J 2001;20:230–3. 11224853 10.1097/00006454-200102000-00027

[2] Enzenauer RW Yamaoka RM Eosinophilic meningitis and hydrocephalus in an infant. Arch Neurol 1982;39:380–1. 7092618 10.1001/archneur.1982.00510180058016

[3] Kuberski T Bart RD Briley JM Recovery of *Angiostrongylus cantonensis* from cerebrospinal fluid of a child with eosinophilic meningitis. J Clin Microbiol 1979;9:629–31. 479360 10.1128/jcm.9.5.629-631.1979 PMC275361

[4] Lindo JF Escoffery CT Reid B Fatal autochthonous eosinophilic meningitis in a Jamaican child caused by *Angiostrongylus cantonensis*. Am J Trop Med Hyg 2004;70:425–8. 15100458

[5] Cooke-Yarborough CM Kornberg AJ Hogg GG A fatal case of angiostrongyliasis in an 11-month-old infant. Med J Aust 1999;170:541–3. 10397046 10.5694/j.1326-5377.1999.tb127880.x

[6] Hu X Du J Tong C Epidemic status of *Angiostrongylus cantonensis* in Hainan island, China. Asian Pac J Trop Med 2011;4:275–7. 21771469 10.1016/S1995-7645(11)60085-0

[7] Wang QP Lai DH Zhu XQ Human angiostrongyliasis. Lancet Infect Dis 2008;8:621–30. 18922484 10.1016/S1473-3099(08)70229-9

[8] Sawanyawisuth K Sawanyawisuth K Intapan PM Specificity of immunoblotting analyses in eosinophilic meningitis. Mem Inst Oswaldo Cruz 2011;106:570–2. 21894378 10.1590/s0074-02762011000500009

[9] Sawanyawisuth K Sawanyawisuth K Senthong V How can clinicians ensure the diagnosis of meningitic angiostrongyliasis? Vector Borne Zoonotic Dis 2012;12:73–5. 21923259 10.1089/vbz.2011.0711

[10] Sawanyawisuth K Sawanyawisuth K Treatment of angiostrongyliasis. Trans R Soc Trop Med Hyg 2008;102:990–6. 18501934 10.1016/j.trstmh.2008.04.021

